# Tripodal Quinone-Cyanine G-Quadruplex Ligands as Novel Photosensitizers on Photoinduced Cancer Cell Death

**DOI:** 10.3390/molecules29215094

**Published:** 2024-10-28

**Authors:** Junya Muramoto, Takashi Sakamoto

**Affiliations:** 1 Graduate School of Systems Engineering, Wakayama University, 930 Sakaedani, Wakayama 640-8510, Japan; muramoto.junya@g.wakayama-u.jp; 2 Faculty of Systems Engineering, Wakayama University, 930 Sakaedani, Wakayama 640-8510, Japan

**Keywords:** guanine-quadruplex, G4 ligand, reactive oxygen species, photosensitizers, apoptosis

## Abstract

Guanine-quadruplex (G4) selective photosensitizers have huge potential for photodynamic therapy against various diseases correlated with G4 DNA and G4 RNAs; however, the types of photosensitizer skeletons available are limited. Herein, we investigated the ability of our original G4 ligands, tripodal quinone-cyanine dyes (tpQCy(s)), which were developed as fluorescent probes for G4, to act as photosensitizers for cancer-selective apoptosis inducers. The results indicated that the tpQCy skeleton has great potential for developing G4-targeted cancer-selective photosensitizers for photodynamic therapy. Among the two tpQCys, only QCy(BnBT)_3_, which has greater G4 selectivity, exhibited photoinduced cytotoxicity in HeLa cell growth, suggesting that the direct oxidation of G4 DNA or RNA is crucial for photoinduced cytotoxicity. RNA-seq analysis using a next-generation sequencing technique revealed that apoptosis was clearly induced by photoirradiation after QCy(BnBT)_3_ treatment.

## 1. Introduction

Among the various non-canonical nucleic acid structures, guanine-quadruplex (G4) structures are expected as novel drug targets because of their existence on the promoter region of oncogenes [1,2,3] and on the telomere region [3,4], which can be extended to various cancer cells. To regulate G4 functions, various G4-specific small-molecular ligands have been developed [5,6,7]. Using these ligands, the regulation of G4 functions was attempted; however, the selectivity and efficiency of G4 regulation remained insufficient. To overcome this problem, some photoresponsive G4-specific small-molecular ligands have been reported, such as porphyrin derivatives [8,9,10,11,12], phthalocyanine derivatives [13,14], dibenzothioxanthene imide [15], and benzothiazolium acetonitrile-methylacridinium π-conjugate [16]. Since almost all of the photoresponsive ligands mentioned above can generate reactive oxygen species (ROS) upon photoirradiation, these ligands could be good photosensitizers that can induce cytotoxicity against only irradiated cells. These G4-targeted ligands are expected to be photosensitizers in photodynamic therapy (PDT) for G4-positive (e.g., telomerase-positive) cancers with extremely low side effects; however, the types of photosensitizer skeletons available remain limited. The poor variation in the skeleton prevents the selective binding toward G4s having different morphology and averts morphology selective regulation of G4’s function. A wide variety in the G4 targeted photosensitizer skeleton would accelerate the development of G4 targeted PDT.

In our recent study, fluorescent G4 ligands, tripodal quinone-cyanine dyes (tpQCy(s)), that can detect G4 nucleic acids with a fluorescence signal turn-on were successfully developed [17,18]. Owing to the large fluorescence enhancement and near-infrared fluorescence properties of the tpQCys, G4 nucleic acids can be clearly imaged in living cells; however, the fluorescence quantum yields of tpQCys were ca. 5%. These lower fluorescence quantum yields indicate that photoactivated tpQCy can generate ROS that strongly oxidize the molecules around tpQCy. Therefore, tpQCy is expected to be a novel photosensitizer skeleton in the PDT of G4-positive cancers. Herein, we describe the availability of the G4 ligand tpQCys as a photosensitizer for G4-targeted cancer PDT.

## 2. Results and Discussion

### 2.1. Photoinduced HeLa Cell Death

**Figure 1:**
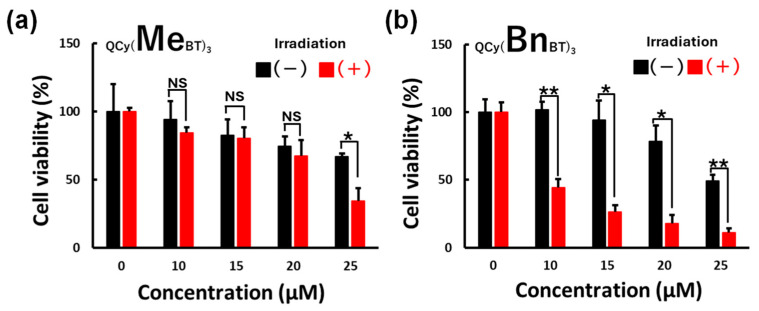
Photoinduced cytotoxicity of QCy(MeBT)_3_ (**a**) and QCy(BnBT)_3_ (**b**) on HeLa cells. Photoirradiation: 530 nm, 0.87 mW/cm^2^, 120 min. For statistical significance, an unpaired *t*-test was performed. *: *p* < 0.01, **: *p* < 0.001, NS: not significant (*p* > 0.05). The experiments were triplicated, and the data are presented as the means ± SD from three independent experiments.

To evaluate the photoinduced cytotoxicity of tpQCys, cell viability after tpQCys treatment and photoirradiation (530 nm) was evaluated. In the case of QCy(MeBT)_3_ (Figure 1a), irradiation-independent but dose-dependent cell death was observed under almost all conditions of QCy(MeBT)_3_ treatment. On the other hand, both dose- and irradiation-dependent cell death was observed in the case of QCy(BnBT)_3_ (Figure 1b). These results indicate that some species generated by the photoirradiation of tpQCys caused cell death with, as expected, ROS being the species most likely to do so. The difference observed in the responses to photoirradiation suggests that the binding selectivity of these two tpQCys for the secondary structure of nucleic acids significantly affects cytotoxicity, i.e., QCy(MeBT)_3_ can bind both with dsDNA and G4 DNA/RNA, whereas QCy(BnBT)_3_ can bind only with G4 DNA/RNA.

### 2.2. Photoinduced ROS Generation

**Figure 2:**
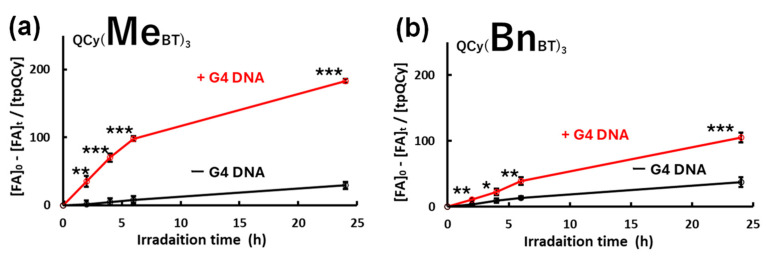
Photoinduced ROS generation abilities of QCy(MeBT)_3_ (**a**) and QCy(BnBT)_3_ (**b**) in the presence or absence of G4 DNA, MycG4. [tpQCy] = 10 µM, [MycG4] = 10 µM, [furfuryl alcohol (FA)] = 2.1 mM, [uridine (internal standard for HPLC analysis)] = 1.5 mM in 50 mM Tris-HCl (pH 7.5) containing 100 mM KCl. Photoirradiation: 530 nm, 0.87 mW/cm^2^, 120 min. For statistical significance, an unpaired *t*-test was performed. *: *p* < 0.05, **: *p* < 0.01, ***: *p* < 0.001. The results of the HPLC analysis for quantifying furfuryl alcohol are shown in Figure S2. The experiments were triplicated, and the data are presented as the means ± SD from three independent experiments.

Encouraged by the above results, the ROS generation abilities of tpQCys were assessed in test tubes using furfuryl alcohol as a singlet oxygen indicator (Figure 2). Results indicated that QCy(MeBT)_3_ and QCy(BnBT)_3_ both have photoinduced ROS generation ability in the presence of G4 DNA (MycG4: 5′-TGAGGGTGGGTAGGGTGGGTAA-3′). In the absence of G4 DNA, the amount of singlet oxygen generated was less than 50% of that generated in the presence of G4 DNA, suggesting that the binding of tpQCys with G4 DNA enhances the ability to generate singlet oxygen. The photoinduced singlet oxygen-generation ability of tpQCys was also enhanced in a high-viscosity environment (Figure S1), where the restriction of the intramolecular rotation of tpQCys enhanced the efficiency of the photoinduced singlet oxygen generation.

**Figure 3:**
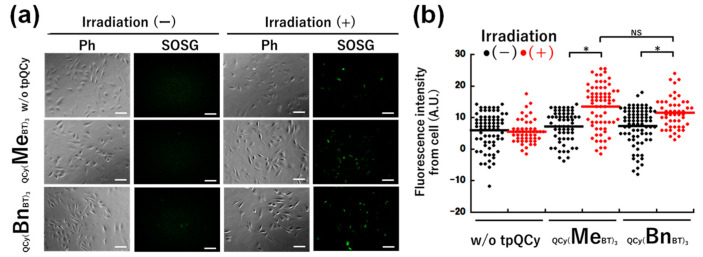
Fluorescence microscopic images (**a**) and fluorescence dot plot analysis (**b**) of SOSG- and tpQCy-treated HeLa cells before and after the photoirradiation. [tpQCy] = 15 µM in 5% glucose, [SOSG] = 15 µM in PBS. Photoirradiation: 530 nm, 0.87 mW/cm^2^, 30 min. Scale bar = 100 µm. For statistical significance, an unpaired *t*-test was performed. *: *p* < 0.0001, NS: not significant (*p* > 0.05).

To assess the photoinduced ROS generation ability of tpQCys in cells, photoirradiation was performed on HeLa cells treated with SOSG (fluorescent singlet oxygen indicator) and tpQCy. As shown in Figure 3a, green SOSG fluorescence in the cells was increased clearly in both cases of tpQCys after photoirradiation, even though the SOSG signal possibly being suppressed because of the very short lifetime of ROS (µsec order) compared with the diffusion constant of molecules in cell (msec order). Together with the results of the dot plot analysis (Figure 3b), the tpQCys generated singlet oxygens in not only a test tube but also in cells. These results suggest that singlet oxygen generation is a major factor in photoinduced cytotoxicity.

### 2.3. G4 Selective Photoinduced DNA Damage

When comparing the abilities of singlet oxygen generation and the photoinduced cytotoxicities of each tpQCy, there appears to be no correlation between these two factors. In fact, QCy(MeBT)_3_, which has a higher ability to generate singlet oxygen (Figure 2), exhibits lower photoinduced cytotoxicity (Figure 1). This strongly suggests that factors other than singlet oxygen generation contribute to photoinduced cytotoxicity besides singlet oxygen generation. When taking into account the binding selectivity of tpQCys, i.e., QCy(MeBT)_3_ can bind both with dsDNA and G4 DNA/RNA but QCy(BnBT)_3_ can bind only with G4 DNA/RNA, it seems that the binding of tpQCys on G4 DNA/RNA is a critical factor in photoinduced cytotoxicity.

**Figure 4:**
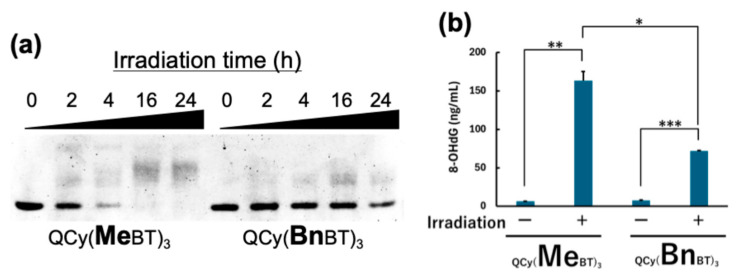
(**a**) Electrophoresis with 18% native polyacrylamide gel. [tpQCy] = 0.5 µM, [MycG4] = 0.5 µM in 50 mM Tris-HCl (pH 7.5) containing 100 mM KCl. Photoirradiation: 530 nm (0.87 mW/cm^2^). (**b**) Quantification of oxidized guanine bases on the MycG4 oligonucleotide. [MycG4] = 5 µM ([guanine] = 14 µg/mL), [tpQCy] = 5 µM. Photoirradiation: 530 nm, 0.87 mW/cm^2^, 24 h. The amounts of 8-OHdG included in the digested nucleoside mixture of MycG4 were quantified by competitive ELISA. For statistical significance, an unpaired *t*-test was performed. *: *p* < 0.001, **: *p* < 0.0001, ***: *p* < 0.0000001. The experiments were triplicated, and the data are presented as the means ± SD from three independent experiments.

To test this hypothesis, the direct damage to G4 DNA caused by photoactivated tpQCys was tested by native polyacrylamide gel electrophoresis. As shown in Figure 4a, photoirradiation decreased the band intensity of intact MycG4 DNA and caused the appearance of a novel band having lower mobility than that of intact MycG4 DNA. This indicates that the increase in molecular volume, i.e., disruption of the G4 structure, was caused by photoirradiation. Since the oxidation of the guanine base on G4 DNA destabilizes the G4 structure [19], and the oxidized guanine base, 8-OHdG, was detected only after photoirradiation (Figure 4b), the results suggest that tpQCys oxidize guanine bases on DNA, particularly at G4 structures. Indeed, the fact that the addition of dsDNA with the AT-rich sequence, which is the binding sequence of QCy(MeBT)_3_, suppressed the production of the delayed band generated by the photoactivated QCy(MeBT)_3_ (Figure S4) strongly suggests that the oxidation ability of QCy(MeBT)_3_ might be suppressed by the presence of genomic DNAs in cells.

### 2.4. Insight of the Photoinduced HeLa Cell Death

**Figure 5:**
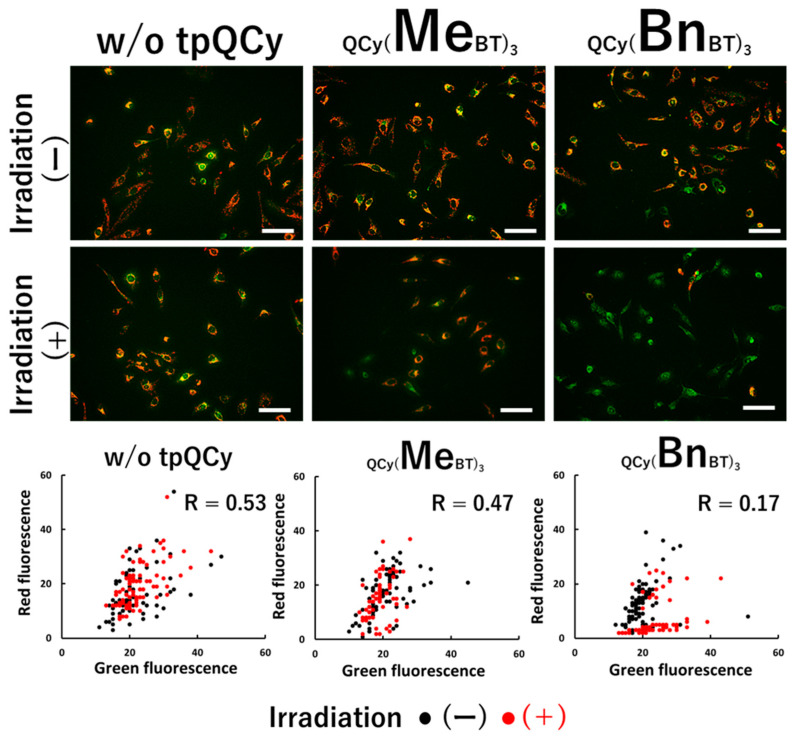
Photoinduced dysfunction of mitochondria in tpQCys-treated HeLa cells. [tpQCy] = 15 µM in 5% glucose, [JC-1] = 15 µM in DMEM (10% FBS). Photoirradiation: 530 nm, 0.87 mW/cm^2^, 120 min, Scale bar = 100 µm.

To gain insight into photoinduced HeLa cell death, we assessed the disruption of the mitochondrial membrane potential, an early apoptotic indicator, before and after photoirradiation using JC-1, whose fluorescence color changed with the disruption of the mitochondrial membrane potential. As shown in Figure 5, a significant red-to-green fluorescence color change was observed only after the photoirradiation of the QCy(BnBT)_3_-treated cells. In the case of the QCy(MeBT)_3_-treated cells, no significant change in fluorescence color was observed, indicating that the apoptosis pathway was not activated by the photoactivated QCy(MeBT)_3_, whereas tpQCys both exhibited ROS generation ability in the cells (Figure 3). These results also suggest that the direct oxidation of the G4 guanine base and disruption of the G4 structure are the key mechanisms underlying photoinduced cell death.

To determine which of the endogenous G4 oxidation pathways contributed to the photoinduced cell death caused by QCy(BnBT)_3_, the cellular distribution of QCy(BnBT)_3_ was investigated by co-staining with MitoBright LT Green, a mitochondria-specific fluorescent probe. As shown in Figure S5, fluorescence signals from QCy(BnBT)_3_ and MitoBright LT Green did not colocalize with each other, indicating that QCy(BnBT)_3_ did not oxidize G4s in mitochondrial DNA. In other words, the major factor underlying photoinduced cell death is the oxidation of G4s on genomic DNA or G4 RNAs.

### 2.5. RNA-Seq Analysis of Photoirradiated HeLa Cells

**Figure 6:**
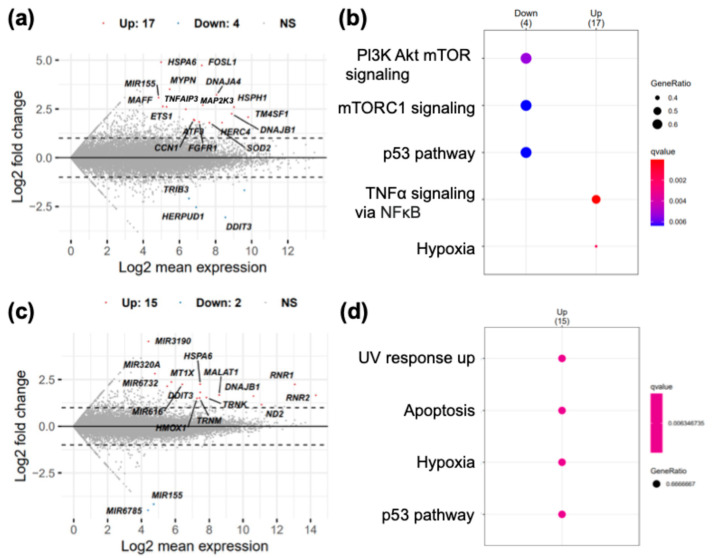
RNA-seq analysis. MA plot (**a**) and enrichment analysis of differentially expressed genes (DEGs) (**b**) of the gene expression in QCy(BnBT)_3_-treated and untreated cells. MA plot (**c**) and enrichment analysis of DEGs (**d**) of the gene expression in irradiated and unirradiated cells. Enrichment analyses were performed using the MSigDB hallmark gene set.

To clarify the impact of QCy(BnBT)_3_ treatment and photoirradiation on transcript levels in cells, RNA-seq analyses were performed before and after QCy(BnBT)_3_ treatment and photoirradiation. As shown in Figure 6a, significant increases in 16 and a decrease in 4 different mRNAs were observed after QCy(BnBT)_3_ treatment. Since 18 of the 20 mRNAs drastically changed by the addition of QCy(BnBT)_3_ have the G4 structure in their RNA strands (Table S1) and lack the common transcription factors that regulate these mRNAs, there is the possibility that the interaction between these G4s and QCy(BnBT)_3_ affected the stability of these mRNAs. Pathway analysis using the MSigDB hallmark gene set (Figure 6b) revealed a downregulation of the cell growth signal (PI3K Akt mTOR signal, mTORC signal), apoptosis accelerator (p53 pathway), and upregulation of the TNFα pathway, which are largely correlated with inflammation. These results suggest that QCy(BnBT)_3_ enhances the response to chemical stress.

Contrary to the case without photoirradiation, as mentioned above, photoirradiation caused a significant increase in nine non-coding RNAs (ncRNA(s)), including two rRNAs (RNR1 and RNR2) and two tRNAs (TRNK and TRNM), among the 15 transcripts. Because these RNAs do not include the G4 structure (Table S2), their increase might be regulated at the transcriptional level. The increment in only six different mRNAs, including four mRNAs having the G4 structure (Figure 6c, HSPA6, DNAJB1, DDIT3, HMOX1), and the enrichment analysis using the MSigDB hallmark gene set (Figure 6d) show that photoirradiation after QCy(BnBT)_3_ treatment caused an upregulation of the apoptosis, p53, and hypoxia pathways, suggesting that photoirradiation caused a decrease in oxygen in cells and apoptosis induction. Because the early apoptotic marker, i.e., depolarization of the mitochondrial membrane, was also induced by photoirradiation (Figure 5), we conclude that cell death was caused by photoinduced ^1^O_2_ generation and enhanced apoptosis signaling. Furthermore, differentially expressed genes related with other programmed cell death, such as necroptosis and ferroptosis, were not detected. Together with the increase in apoptosis-related genes observed after photoirradiation, we conclude that apoptosis is the major mechanism underlying the photoinduced cytotoxicity.

### 2.6. Cancer-Selective Cell Death

**Figure 7:**
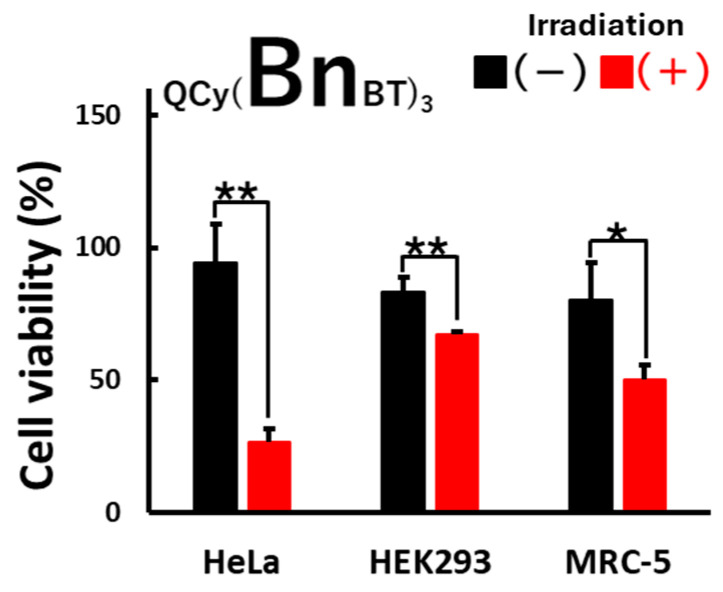
Photoinduced cytotoxicity of tpQCys on HeLa, HEK293, and MRC-5 cells. [tpQCy] = 15 µM in 5% glucose. Photoirradiation: 530 nm, 0.87 mW/cm^2^, 120 min. For statistical significance, unpaired *t*-tests were performed. *: *p* < 0.05, **: *p* < 0.01. The experiments were triplicated, and the data are presented as the means ± SD from three independent experiments.

Finally, the selectivity for photoinduced cytotoxicity in cancer cells was assessed using human embryonic kidney (HEK293) and lung fibroblast (MRC-5) cells as normal cell strains. As shown in Figure 7, the photoinduced cytotoxicity of QCy(BnBT)_3_ in HeLa cells was much greater than that observed in HEK293 and MRC-5 cells. These results suggest that the photoinduced cytotoxicity of QCy(BnBT)_3_ is selective for cancer. When taking into account the telomerase activity of these three cell lines, HeLa > HEK293 >> MRC-5 [20,21], the cancer selectivity observed cannot be explained only by the binding of QCy(BnBT)_3_ to the telomere regions of the chromosomes. Together with the relatively homogenous intercellular distribution of QCy(BnBT)_3_ (Figure S5), QCy(BnBT)_3_ appears to act on genomic G4 DNA or G4 RNAs. As an off-target effect, QCy(BnBT)_3_ is cytotoxic in the absence of photoirradiation. This decreases the photosensitivity of the photoinduced cytotoxicity. Further improvement of the tpQCy structure, e.g., the introduction of heavy atoms and/or expansion of π-conjugation length, might enable low-concentration treatment and a decrease in the off-target effect.

## 3. Materials and Methods

### 3.1. Materials

tpQCys were synthesized by methods found in the literature [17,18]. Briefly, corresponding 2-methyl-*N*-alkylbenzothiazolium halide was condensed with 2-Hydroxy-1,3,5-benzenetricarbaldehyde by Knoevenagel condensation and then purified with reversed-phase HPLC. SOSG and SYBR^®^ Gold were purchased from ThermoFisher Scientific K. K. (Tokyo, Japan). The JC-1 MitoMP Detection Kit, MitoBright LT Green, and Cell Counting Kit-8 (CCK-8) were purchased from Dojindo Laboratories (Kumamoto, Japan). A New 8-OHdG Check ELISA kit for 8-OHdG detection was purchased from Nikken Seil (Tokyo, Japan). NucleoSpin^®^ RNA and alkaline phosphatase were purchased from Takara Bio Inc. (Shiga, Japan) Oligodeoxyribonucleotides were purchased from Macrogen (Tokyo, Japan). Cell lines were provided by the RIKEN BRC through the National BioResource Project of the MEXT/AMED, Japan. The other reagents were purchased from the Fujifilm Wako Pure Chemical Co., Ltd. (Osaka, Japan) or Tokyo Chemical Industry Co., Ltd. (Tokyo, Japan).

### 3.2. Cell Culture, tpQCy Treatment, and Photoirradiation

A suspension of HeLa cells in D-MEM (10% FBS) was seeded in the wells of a 96-well culture plate (1.8 × 10^4^ cells/well) and cultured for 24 h in a humidified chamber (5% CO_2_, 37 °C). Cells were treated with tpQCy (in 5% glucose aq. soln.), incubated for 10 min in a humidified chamber (5% CO_2_, 37 °C), washed with growth medium, and then irradiated for 2 h using an UltraBright LED transilluminator (BMTLL-300, BM Equipment Co., Ltd. Tokyo, Japan; 530 nm, 0.87 mW/cm^2^).

### 3.3. Cell Viability

Cells were cultured for 24 h on 96-well culture plates, treated with tpQCy (in 5% glucose aq. soln.) for 10 min, irradiated (530 nm, 0.87 mW/cm^2^) for 120 min, and then cultured in fresh culture medium for another 24 h. Cell viability was evaluated using CCK-8 according to the manufacturer’s protocol. The OD (450 nm) of each well in the 96-well plate was measured using a microplate reader (MPR-A100, AS ONE, Osaka, Japan).

### 3.4. Singlet Oxygen Detection in the Tube

MycG4 (100 µM), Tris-HCl (250 mM, pH 7.5), and KCl (500 mM) were mixed, heated at 95 °C for 5 min, and allowed to stand at room temperature for 30 min. After 30 min of incubation, the samples were adjusted to MycG4 (10 µM), furfuryl alcohol (2.1 mM), uridine (5 mM), tpQCy (10 µM), Tris-HCl (50 mM, pH 7.5), and KCl (100 mM), and then irradiated (530 nm, 0.87 mW/cm^2^) for 120 min. The samples were then analyzed using a reversed-phase high-performance liquid chromatograph (SPD-20A, SHIMADZU, Kyoto, Japan) equipped with a Cosmosil C18 column (Nacalai Tesque Inc., Kyoto, Japan; 5 µm, 4.6 × 150 mm) in isocratic mode (0.1% TFA/H_2_O).

### 3.5. Fluorescence Microscopy Imaging

Fluorescence microscopic imaging was performed using a fluorescence microscope (BZ-X810, KEYENCE, Kyoto, Japan) equipped with an objective lens (Plan Fluor 20×) and various filter blocks: Ex470/40, DM495, Em525/50 (for SOSG); Ex470/40, DM495, Em525/50 (for green fluorescence of JC-1); Ex545/25, DM565, Em605/70 (for red fluorescence of JC-1); Ex560/40, DM585, Em700/75 (for QCy(MeBT)_3_); Ex560/40, DM585, Em665lp (for QCy(BnBT)_3_); and Ex470/40, DM495, Em525/50 (for MitoBright LT Green).

### 3.6. SOSG Staining

HeLa cells (1.0 × 10^4^ cells) were cultured for 24 h in a 96-well culture plate and then treated with tpQCy (15 µM in 5% glucose aq. soln.) for 10 min and SOSG (15 µM) for 30 min [22]. The cells were washed with 1 × PBS twice and irradiated (530 nm, 0.87 mW/cm^2^) for 30 min before fluorescence microscopic imaging.

### 3.7. JC-1 Staining

HeLa cells (1.8 × 10^4^ cells) were cultured for 24 h in a 96-well culture plate, treated with tpQCy (15 µM in 5% glucose aq. soln.) for 10 min, irradiated (530 nm, 0.87 mW/cm^2^) for 120 min, and cultured in a fresh culture medium for another 4 h before the addition of JC-1 (10 µM). The mitochondrial membrane potential of the cells was evaluated using a JC-1 according to the manufacturer’s protocol.

### 3.8. MitoBright LT Green Staining

HeLa cells (1.1 × 10^4^ cells) were cultured for 24 h in a 96-well culture plate and treated with tpQCy (15 µM in 5% glucose aq. soln.) for 30 min, and then MitoBright LT Green (0.1 µM) was added and incubated for 30 min before fluorescence microscopic imaging.

### 3.9. Native Polyacrylamide Gel Electrophoresis

MycG4 (100 µM), Tris-HCl (250 mM, pH 7.5), and KCl (500 mM) as the buffer were mixed, heated at 95 °C for 5 min, and allowed to stand at room temperature for 30 min. The samples were adjusted to MycG4 (10 µM), tpQCy (10 µM), Tris-HCl (50 mM, pH 7.5), and KCl (100 mM) concentrations and then irradiated (530 nm, 0.87 mW/cm^2^) for 24 h. The samples containing DNA (1.0 pmol) were then analyzed by electrophoresis in 18% polyacrylamide gel at 0 °C. Subsequently, the gels were stained with SYBR^®^ Gold and imaged using a gel-imager (FAS-BG LED BOX, Nippon Genetics, Co. Ltd., Tokyo, Japan).

### 3.10. Detection of Oxidized Guanine

tpQCy (10 µM) with MycG4 (10 µM) was irradiated (530 nm, 0.87 mW/cm^2^) for 24 h and treated with Nuclease P1 (50 U) (22 h, 50 °C) and alkaline phosphatase (40 U, 23 h, 37 °C) [23]. The 8-OH-dG level of MycG4 was evaluated using a New 8-OHdG Check according to the manufacturer’s protocol. The OD (450 nm) of each well on a 96-well plate was measured using a microplate reader (MPR-A100, ASONE, Osaka, Japan).

### 3.11. RNA-Seq on NGS Platforms

HeLa cells (2.5 × 10^5^ cells) were cultured for 48 h in 60 mm dishes, treated with QCy(BnBT)_3_ (15 µM in 5% glucose aq. soln.) for 10 min, irradiated for 120 min (530 nm, 0.87 mW/cm^2^), and cultured in fresh culture medium for another 4 h. Cells without irradiation and QCy(BnBT)_3_ were used as controls. Total RNA was extracted using NucleoSpin^®^ RNA and shipped to an NGS analysis service provider (Bioengineering Lab., Sagamihara, Japan). Differentially expressed genes were analyzed using RNAseqChef [24].

## 4. Conclusions

Our two tpQCy G4 ligands (QCy(MeBT)_3_ and QCy(BnBT)_3_) both generate ^1^O_2_ upon 530 nm photoirradiation, but only QCy(BnBT)_3_ exhibits cancer-selective photoinduced cytotoxicity. A comparison of the photoinduced cytotoxicity and apoptosis induction abilities of the two tpQCys revealed that the G4 selectivity of the tpQCy ligands is critical for photoinduced cell death. Together with the fact that the oxidation of the guanine base on the G4 DNA was clearly induced by photoirradiation after tpQCy treatment, the oxidation of the guanine base on G4 might be a key phenomenon for explaining the photoinduced cytotoxicity of QCy(BnBT)_3_. Given the relatively homogeneous intracellular distribution of QCy(BnBT)_3_ and the lower correlation between photoinduced cytotoxicity and telomerase activity in cells, the damage induced by photoactivated QCy(BnBT)_3_ on genomic G4 DNA or G4 RNAs might be a major factor in the cancer-selective photoinduced cytotoxicity of QCy(BnBT)_3_. The results clearly indicated that QCy(BnBT)_3_ can function as a photosensitizer for cancer-selective cell death via photoinduced apoptosis induction and that tpQCy can be used as a new type of photosensitizer skeleton. Riboflavin is one of the classical photosensitizers for PDT. The potential of riboflavin and its derivatives in PDT is high [25]. However, riboflavin requires UV or blue light excitation, which cannot penetrate the bio-organs. This prevents the in vivo use of the deep parts of the bio-organs. Furthermore, riboflavin does not act as a specific ligand of any biomolecule. This decreases the specificity of cancer treatments. Our ligand with a tpQCy skeleton can be excited at longer wavelengths and exhibits target selectivity. Therefore, we conclude that the tpQCy skeleton has advantages over riboflavin for cancer-selective PDT. Since the three benzothiazole parts of tpQCy can be easily changed because of its simple synthetic protocol, further development of the photosensitizers based on the tpQCy skeleton may enable near-infrared excitation of the photosensitizers and selective morphology-specific G4 regulation.

## Data Availability

The data presented in this study are available on request from the corresponding author. The RNA-seq raw data reported in this paper have been deposited in DDBJ database under accession number PRJDB19000.

## Supplementary Materials

The following supporting information can be downloaded at https://www.mdpi.com/article/10.3390/molecules29215094/s1: Figure S1: Photoinduced ROS generation abilities of tpQCys in the presence or absence of 50% glycerol in H_2_O; Figure S2: HPLC analysis for quantifying furfuryl alcohol; Figure S3: Photoinduced ROS generation abilities of tpQCys in the presence or absence of G4 DNA; Figure S4: PAGE analysis of the photooxidation-induced structural change in MycG4 DNA in the presence or the absence of AT-rich dsDNA; Figure S5: Colocalization analysis of fluorescence microscopic images of living HeLa cells after co-staining with MitoBright LT Green and tpQCys; and Figure S6: Volcano plots of differentially expressed genes. Table S1. Differentially expressed genes in the HeLa cells after the QCy(BnBT)_3_ treatment. Table S2. Differentially expressed genes in the QCy(BnBT)_3_ treated HeLa cells after the photoirradiation.
