# Supplementary material for: Tripodal Quinone-Cyanine G-Quadruplex Ligands as Novel Photosensitizers on Photoinduced Cancer Cell Death
Source: Molecules. 2024 Oct 28;29(21):5094. doi: 10.3390/molecules29215094 (PMC11547667; doi:10.3390/molecules29215094)
Supplement: Supplementary file 1 [file molecules-29-05094-s001.zip › molecules-3255625-supplementary.pdf]

Supporting Information

Tripodal quinone-cyanine G-quadruplex ligands as novel photosensitizers on photo-induced cancer cell death

Junya Muramoto [1] and Takashi Sakamoto [1,2,*]

[1]Graduate School of Systems Engineering, Wakayama University, 930 Sakaedani, Wakayama 640-8510, Japan; e-mail@e-mail.com

[2]Faculty of Systems Engineering, Wakayama University, 930 Sakaedani, Wakayama 640-8510, Japan; e-mail@e-mail.com

*Correspondence: tsakamo@wakayama-u.ac.jp

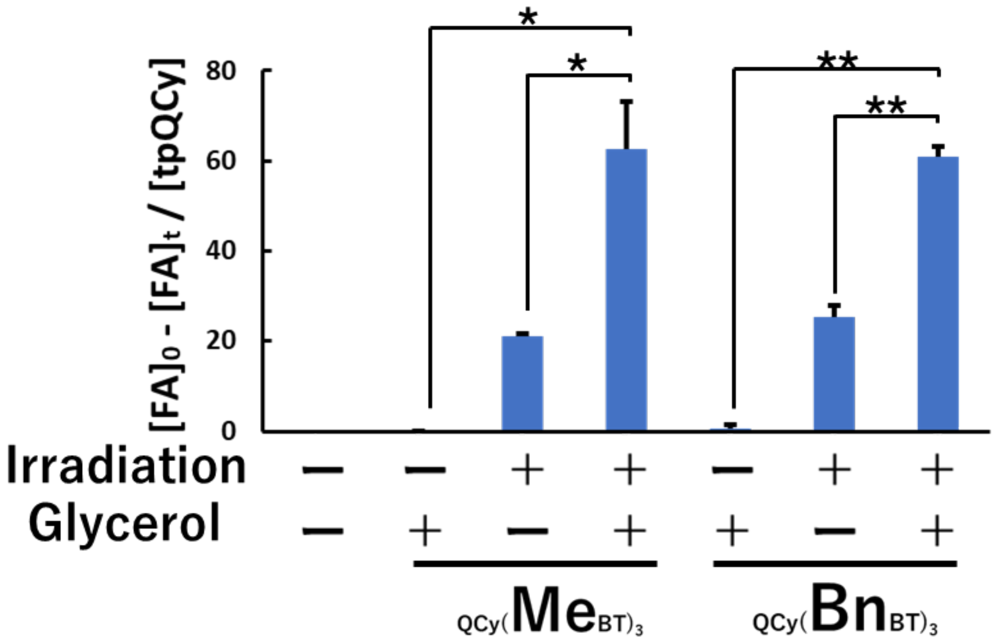

Figure S1. Photo-induced ROS generation abilities of tpQCys in the presence or absence of 50% glycerol in H_2O. [tpQCy] = 10 μM, [Furfuryl alcohol (FA)] = 2.1 mM, [Uridine (internal standard for HPLC analysis)] = 1.5 mM. Photoirradiation: 530 nm, 0.87 mW/cm^2, 120 min. For statistical significance, an unpaired t-test was performed. . $^*p < 0.01$,** $p < 0.001$. The experiments were triplicated, and the data are presented as the means ± SD from three independent experiments.

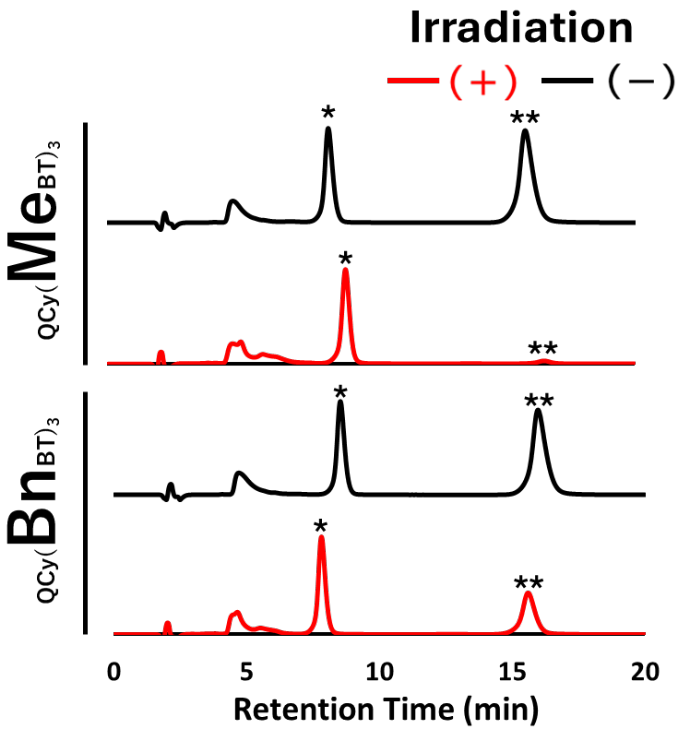

Figure S2. HPLC analysis for quantifying furfuryl alcohol. [tpQCy] = 10 μM, [MycG4] = 10 μM, [Furfuryl Alcohol] = 2.1 mM, [Uridine] = 1.5 mM in Tris-HCl (pH7.5) containing 100 mM KCl. Photoirradiation：530 nm, 0.87 mW/cm^2, 24 h. Isocratic: H_2O with 0.1% TFA. Flow rate: 1.0 mL / min. Detection: 215 nm. Injection: 40 μL. "*" and "**" indicate the peaks identical to uridine and furfuryl alcohol, respectively. MycG4: 5'-TGAGGGTGGGTAGGGTGGGTAA-3'.

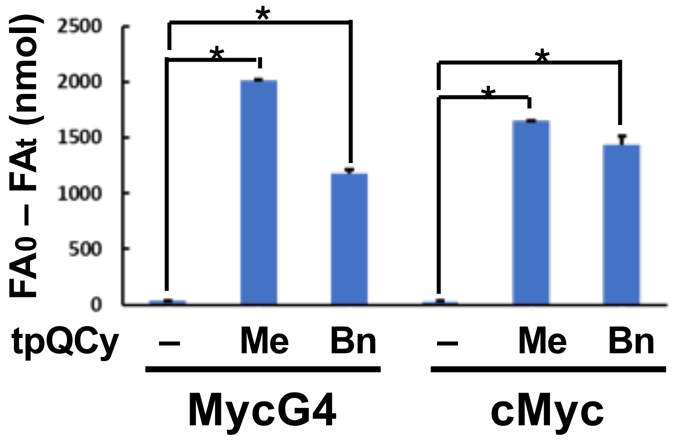

Figure S3. Photo-induced ROS generation abilities of tpQCys in the presence or absence of G4 DNA. [tpQCy] = 10 μM, [G4 DNA (MycG4 or cMyc)] = 10 μM, [Furfuryl alcohol (FA)] = 2.1 mM, [Uridine (internal standard for HPLC analysis)] = 1.5 mM in 50 mM Tris-HCl (pH 7.5) containing 100 mM KCl. Photoirradiation: 530 nm, 0.87 mW/cm^2, 120 min. For statistical significance, an unpaired t-test was performed. *: $p < 0.00001$. The experiments were triplicated, and the data are presented as the means ± SD from three independent experiments. MycG4: 5′-TGAGGGTGGGTAGGGTGGGTAA-3′; cMyc: 5′-TGGGGAGGGTGGGGAGGGTGGGGAAGG-3′.

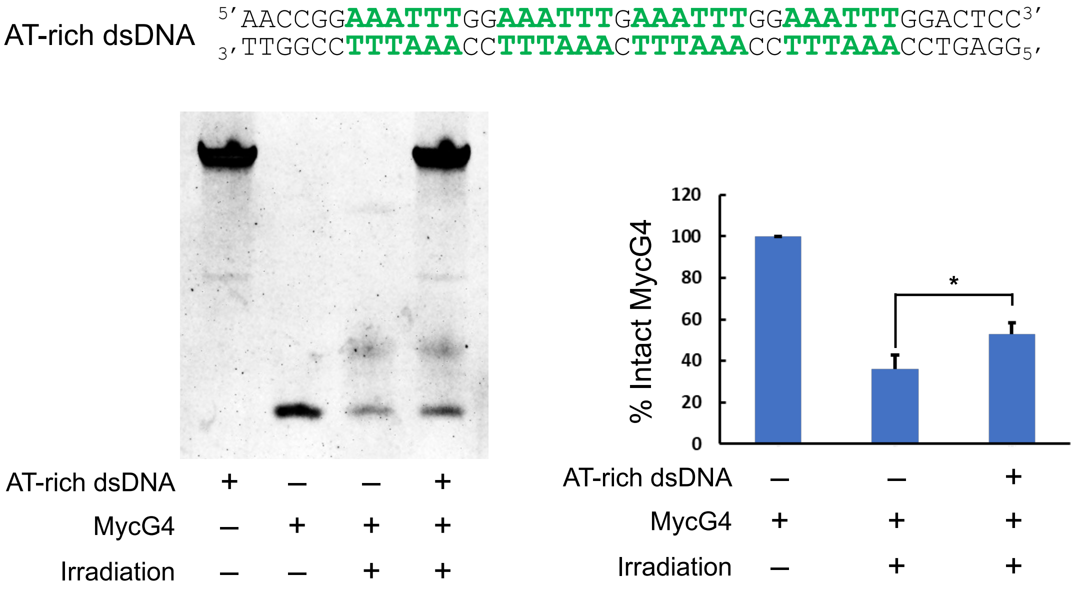

Figure S4. PAGE analysis of the photooxidation induced structural change in MycG4 DNA in the presence or the absence of AT-rich dsDNA. 16% native polyacrylamide gel electrophoresis. [tpQCy] = [AT-rich dsDNA] = [MycG4] = 0.5 μM in 50 mM Tris-HCl (pH 7.5) containing 100 mM KCl. Photoirradiation: 530 nm, 0.87 mW/cm^2, 6 h. For statistical significance, an unpaired t-test was performed. *: $p < 0.05$. The experiments were triplicated, and the data are presented as the means ± SD from three independent experiments.

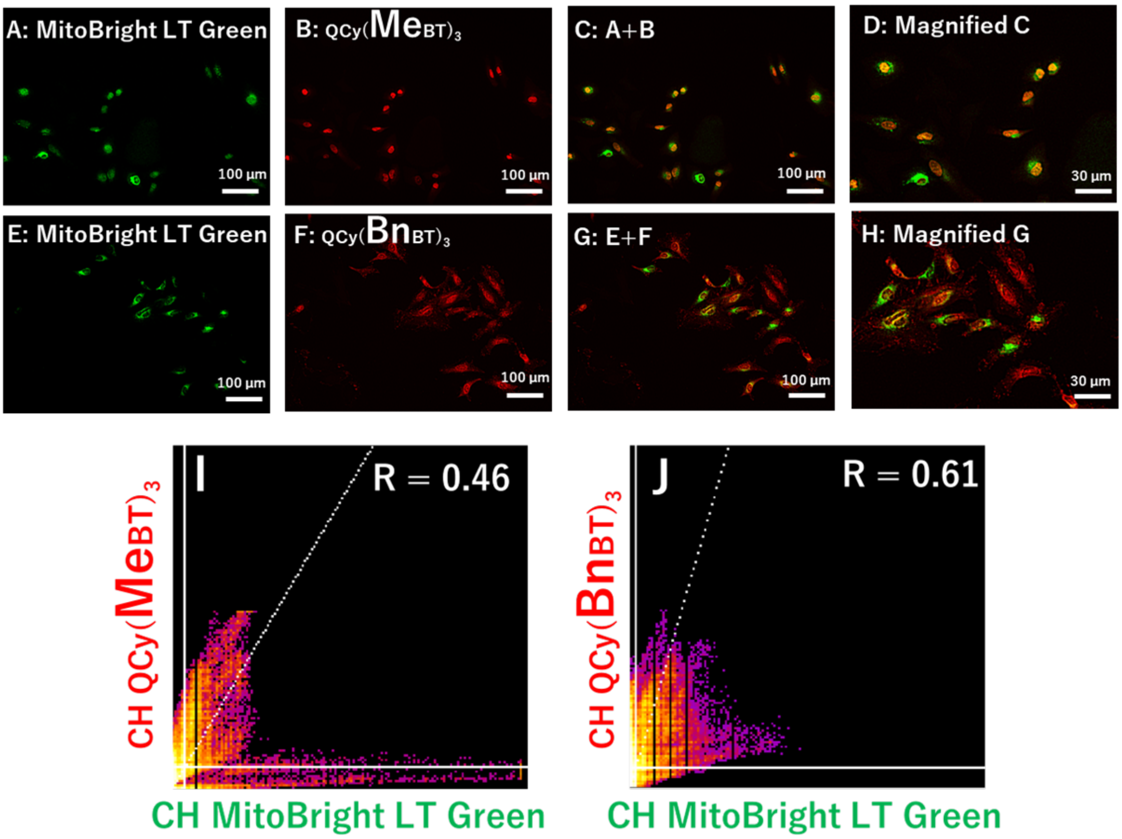

Figure S5. Colocalization analysis of fluorescence microscopic images of living HeLa cells after co-staining with MitoBright LT Green and tpQCys. (A, E) Fluorescence image with 525 nm emission of MitoBright LT Green, (B) Fluorescence image with 700 nm emission of QCy(MeBT)3, (C) merged image of (A,B), (D) magnified image of (C), (F) Fluorescence image with 800 nm emission of QCy(BnBT)3, (G) merged image of (E,F), (H) magnified image of (G), (I,J) Results of the colocalization analyses of C and G. R indicates Pearson's R value. Staining: [tpQCy] = 15 μM in 5% glucose aq. soln, [MitoBright LT Green] = 0.1 μM in DMEM(10% FBS).Imaging: Filter blocks: Ex 470/40, DM495, Em 525/50 (for Mito Green); Ex 560/40, DM585, Em 700/75 (for 700 nm); Ex 560/40, DM585, Em 665lp (for 800 nm). Exposure time: 1/3 s(for Mito Green); 1/2 s(for 700 nm); 1/12 s(for 800 nm).

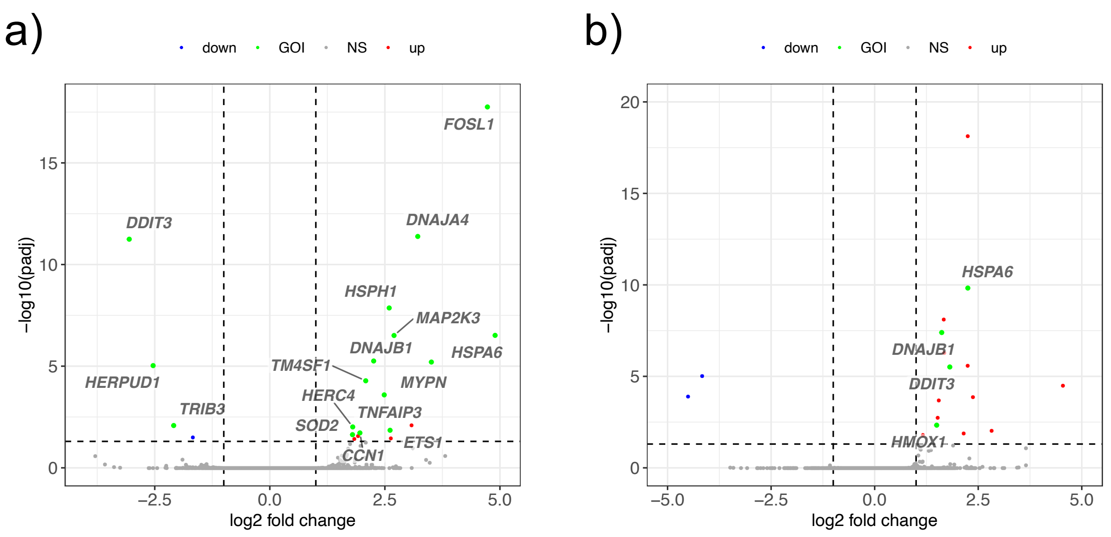

Figure S6. Volcano plots of differentially expressed genes with the QCy(BnBT)$_3$ treatment (a) and with the photoirradiation after the QCy(BnBT)$_3$ treatment (b).

Table S1. Differentially expressed genes in the HeLa cells after the $QCy(BnBT)_3$ treatment.

Gene name	RNA Classification	Secondary structure[a]
FOSL1	mRNA	G4
DNAJA4	mRNA	G4
DDIT3	mRNA	G4
HSPH1	mRNA	G4
HSPA6	mRNA	G4
MAP2K3	mRNA	G4
DNAJB1	mRNA	G4
MYPN	mRNA	G4
HERPUD1	mRNA	G4
TM4SF1	mRNA	G4
TNFAIP3	mRNA	G4
MIR155	ncRNA	–
TRIB3	mRNA	G4
HERC4	mRNA	G4
ETS1	mRNA	G4
CCN1	mRNA	G4
SOD2	mRNA	G4
ATF3	mRNA	–
PDIA4	mRNA	G4
MAFF	mRNA	–
FGFR1	mRNA	G4

[a]To identify G4 structure in the RNAs, G4 database, G4 Atlas (https://www.g4atlas.org) was used.

Table S2. Differentially expressed genes in the QCy(BnBT)$_3$ treated HeLa cells after the photoirradiation.

Gene name	RNA Classification	Secondary structure[a]
RNR1	rRNA	–
HSPA6	mRNA	G4
RNR2	rRNA	–
DNAJB1	mRNA	G4
MALAT1	ncRNA	–
MIR616	Precursor RNA	–
DDIT3	mRNA	G4
MIR155	ncRNA	–
MIR3190	Precursor RNA	–
MIR6785	Precursor RNA	–
MT1X	mRNA	–
TRNK	tRNA	–
TRNM	tRNA	–
HMOX1	mRNA	G4–
MIR320A	Precursor RNA	–
MIR6732	Precursor RNA	–
ND2	mRNA	–

[a]To identify G4 structure in the RNAs, G4 database, G4 Atlas (https://www.g4atlas.org) was used.
